# Expanded molecular evidence of soil-transmitted helminth and *Schistosoma* spp. infections in Myanmar schoolchildren: A qPCR update

**DOI:** 10.1371/journal.pntd.0014384

**Published:** 2026-05-26

**Authors:** Eindra Aung, Natasha Collinson, Kay Thwe Han, Nyein Nyein Hlaing, Moe Moe Aye, Myo Win Htun, Khin Thet Wai, Su Mon Myat, Thida Lay Thwe, Aung Tun, Kinley Wangdi, Yuesheng Li, Gail M. Williams, Archie C. A. Clements, Susana Vaz Nery, Donald P. McManus, Darren J. Gray, Catherine A. Gordon

**Affiliations:** 1 School of Clinical Medicine, University of New South Wales, Sydney, Australia; 2 Pain Management Research Institute, Kolling Institute, Northern Sydney Local Health District, University of Sydney, Sydney, Australia; 3 QIMR Berghofer, Infection and Inflammation, Applied Tropical and Molecular Parasitology, Brisbane, Australia; 4 Parasitology Research Division, Department of Medical Research, Ministry of Health and Sports, ‌‌Yangon, Myanmar; 5 Department of Zoology, University of Yangon, Yangon, Myanmar; 6 Department of Zoology, Maubin University, ‌‌Maubin, Myanmar; 7 Department of Public Health, Ministry of Health and Sports, Nay Pyi Taw, Myanmar; 8 Department of Zoology, Yangon University of Distance Education, Yangon, Myanmar; 9 Ministry of Health and Sports, Nay Pyi Taw, Myanmar; 10 HEAL Global Research Centre, Health Research Institute, Faculty of Health, University of Canberra, Canberra, Australia; 11 Hunan Institute of Parasitic Diseases, World Health Organization Collaborating Centre for Research and Control on Schistosomiasis in Lake Region, Yueyang, China; 12 QIMR Berghofer, Population Health, Global Health and Tropical Medicine, Brisbane, Australia; 13 School of Public Health, University of Queensland, Brisbane, Australia; 14 Queen’s University Belfast, Belfast, Northern Ireland, United Kingdom; 15 The Kirby Institute, University of New South Wales, Sydney, Australia; 16 Center for Tropical Health and Emerging Diseases, Herston, Australia; 17 The University of Queensland, School of Medicine, Brisbane, Australia; George Washington University School of Medicine and Health Sciences, UNITED STATES OF AMERICA

## Abstract

Building on our previous report of high prevalence of soil-transmitted helminth (STH) infections among Myanmar schoolchildren (Aung et al., Infectious Diseases of Poverty, 2022), we conducted additional molecular screening of archival stool samples from the same cohort in Phyu Township, Bago Region, to investigate additional helminth infections. We also report finding of other helminths by Kato-Katz in the previous study that were not previously published. Stool samples utilised in this study were collected in 2016 and the DNA extracted in 2017 and kept stored at -20°C until further molecular characterisation in this study in 2025. Using quantitative PCR (qPCR), we detected *Schistosoma* DNA in two of 264 samples, *Strongyloides stercoralis* DNA in twelve, and *Ancylostoma ceylanicum* in eleven. Although sequencing of the *Schistosoma*-positive samples was unsuccessful, the molecular evidence aligns with other recent reports suggesting emerging or cryptic transmission of schistosomiasis in Myanmar. The epidemiology of schistosomiasis in the region remains poorly defined, highlighting the need for targeted snail surveys, environmental DNA (eDNA) monitoring, and host sampling to confirm transmission foci. This study demonstrates the added value of molecular diagnostics for complementing traditional parasitological methods and guiding surveillance and control strategies in areas of emerging endemicity.

## Author summary

This study builds on earlier work that identified a high prevalence of soil-transmitted helminths (STH) – a group of common intestinal worms, in school students in Myanmar. We re-examined stored stool DNA samples from the same children to look for additional parasite infections. We also report the presence of several other worm species that were observed using standard microscopy methods in the original study but were not reported at that time. Using sensitive DNA-based tests, we identified both *Schistosoma* spp. – a zoonotic blood fluke, and *Strongyloides stercoralis* – an important roundworm of humans, and the zoonotic hookworm *Ancylostoma ceylanicum*. Although we were unable to confirm the exact species of schistosome detected, our findings are consistent with other recent studies and suggests that schistosomiasis may be more widespread in Myanmar than previously recognised. At present, little is known about where and how schistosomiasis is being transmitted in the country. Our results highlight the need for further investigations, including surveys of freshwater snails, testing of water sources using environmental DNA, and sampling of people and animals, to better understand transmission dynamics to support more effective control and prevention strategies.

## Introduction

**Fig 1:**
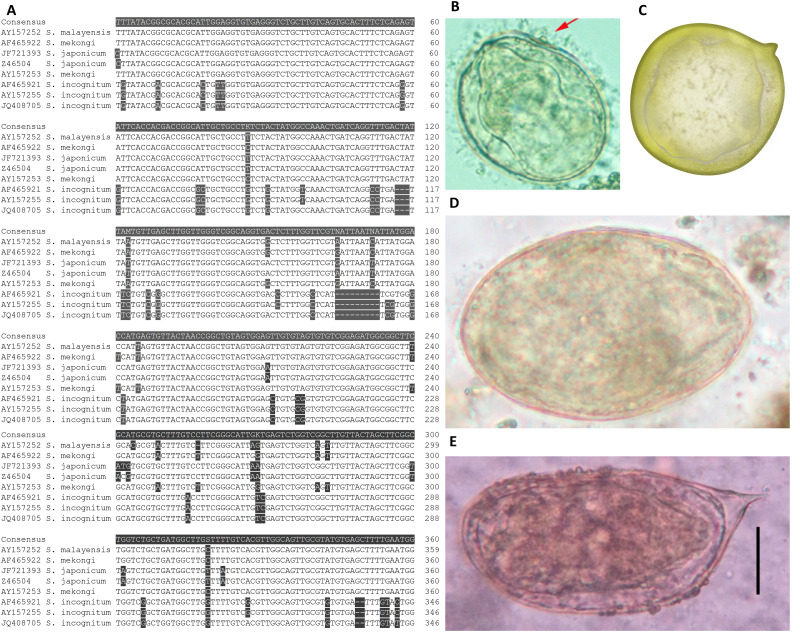
A) Alignment of 28S ribosomal primer binding region [8] for the three Asian schistosome species (*Schistosoma mekongi, S. japonicum,* and *S. malayensis*) showing high genetic similarity. **B)** *S. mekongi* eggs (50 – 80 µm long by 40 – 65 µm wide) and **C)** *S. malayensis* eggs (52 – 90 µm long x 33 – 62 µm wide) are generally smaller and rounder [9] than **D)** *S. japonicum* eggs (70 – 100 µm long by 55 – 64 µm wide), while **E)** *S. incognitum* eggs (85 µm long x 50 µm wide) have a boxy shape with a large terminal spine. Panel B is from the CDC website, panel C is an artistic representation of ‌‌*S. malayensis,* panel D was provided by author CAG, panel E is reproduced from Ajjampur, 2024 [10].

Schistosomiasis is a disease caused by blood flukes of the genus *Schistosoma* which are transmitted by freshwater snails [1,2]. There are three identified species that cause human infections in Asia. *Schistosoma japonicum* has the widest distribution in China, the Philippines, and small pockets of Indonesia. *S. malayensis* is restricted to Malaysia, and *S. mekongi* is present in small areas of Cambodia and Lao PDR, and previously Thailand [1]. All three are zoonotic [3–7]. Morphologically and genetically these species are very similar; it is difficult to differentiate species based on morphology alone (**Fig 1**). The main diagnostic method for schistosomiasis is the microscopic Kato-Katz (KK) technique, which has poor sensitivity at low intensity infections and relies solely on morphology to diagnose parasite infection.

**Fig 2:**
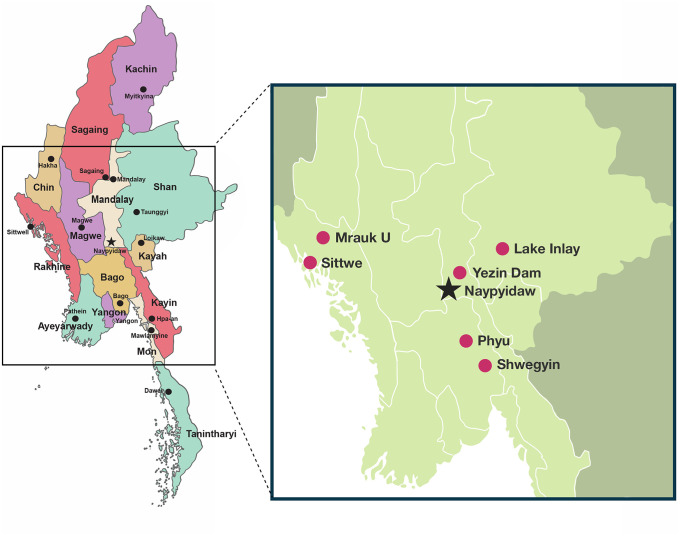
Showing locations of previous schistosomiasis outbreaks in Rakhine State (2018) and Lake Inlay (Inle) in Shan State (2012 and 2013), Shwegyin in Bago Region (2016, 2017), the location of Phyu Township, also in Bago Region, where the current study was performed (2016), and Yezin Dam, site of a snail survey in 2019 – 2020 [17].

Myanmar has been considered non-endemic for schistosomiasis, although there have been unconfirmed historical reports of both *S. japonicum* and *S. mekongi,* with diagnosis based primarily on morphology. Meanwhile there have been several studies over the last 13 years indicating that schistosomiasis is either emerging or re-emerging in the country (**Fig 2**). The first of these studies showed a serological prevalence of 23.8% for schistosomiasis around Lake Inle (Inlay) between 2012 and 2013 [11]. The serological method used, enzyme-linked immunosorbent assay (ELISA) IgG (AccuDiag Schistosoma IgG) does not differentiate between *S. japonicum* and *S. mekongi*. According to the manufacturer’s website the test has a sensitivity of 100% and specificity of 85%, which indicates there may be some cross-reactivity [12]. In 2018 there was an outbreak of schistosomiasis in Rakhine State in Mrauk-U and Sittwe townships with more than 400 cases confirmed and another 800 cases suspected [13]. Diagnosis in that outbreak was via KK. A prevalence of 2.9% (6/205 participants) was identified in 2016 in Shwegyin (Shwe Kyin) Township, Bago region by KK, while 8 samples were positive by conventional Polymerase Chain Reaction (PCR) [14] with bands of 300 bp, associated with *S. mekongi*, identified [15]. A year later in 2017 a *Schistosoma* spp. prevalence of 3% was identified across four villages in Shwegyin across inland and riverside villages (n = 698) by KK (n = 20) and wet mount microscopy (n = 7) [16]. A case study associated with the 2018 outbreak in Rakhine reported schistosomiasis in a patient using an *S. mansoni* ELISA [17]. As *S. mansoni* is restricted to Africa and South America, and not found in Asia, the result almost certainly reflects cross-reactivity with the local *Schistosoma* species responsible for infection. Thus, there remains some uncertainty regarding which *Schistosoma* species are responsible for infections in Myanmar. Historical movements of Japanese troops during World War II and the country’s proximity to endemic areas in Thailand raise the possibility of multiple species infecting humans. However, the detection of a ~ 300 bp amplicon consistent with *S. mekongi* by conventional PCR suggests that *S. mekongi* is the most likely species involved.

*Schistosoma* parasites have a high degree of specificity for their molluscan hosts. Even within the same species complex of *Schistosoma*, certain geographical strains and their associated snail hosts are not compatible and cannot establish infection [18,19]. In Cambodia, Lao PDR, and Thailand *Neotricula aperta* snails are intermediate hosts for *S. mekongi* while *Oncomelania hupensis* snails are hosts of *S. japonicum* in China (*O. h. hupensis*), the Philippines (*O. h. quadrasi*), and Indonesia (*O. h. lindoensis*); in Malaysia *Robertsiella kaporenisis* is the molluscan host*.* There is limited information on snail intermediate hosts or definitive hosts for human schistosomiasis in Myanmar. The ruminant infecting *S. spindale* has been identified in *Indoplanorbis exustus* snails from Yezin Dam, a man-made reservoir located in Zayarthiri township in Nay Pyi Taw City [17] (**Fig 2**). *I. exustus* snails were also identified from Lake Inlay in the same survey. Notably, *I. exustus* is also an intermediate host for *S. incognitum* (**Fig 1**) which has previously been identified in animals from Thailand and Indonesia [20–22], and more recently excreted by two humans from India [10]; this may not be representative of true infection and merely eggs passing through the gastrointestinal tract. *Bithynia siamensis*, the snail host for *Opisthorchis sinensis,* was identified from Lake Inlay and Yezin Dam, along with cercariae of *O. sinensis* [17,23].

Definitive hosts between the three species potentially causing schistosomiasis in Myanmar differ significantly, with both *S. mekongi* and *S. incognitum* identified from dogs, pigs, and rats, as well as sheep and goats for *S. incognitum,* while *S. japonicum* has 46 known mammalian hosts with water buffalo a notable major reservoir host. Experimental infections of primates with *S. incognitum* have been successful, indicating human infection may be possible [10,24,25]. With the high number of potential hosts for *S. japonicum* it is important to confirm which species is occurring in Myanmar and identify any possible animal hosts which would need to be considered as part of a One Health approach to schistosomiasis control.

## Methods

### Ethics statement

Ethical approval was provided by the Protocol and Ethics Review Committee, the Department of Medical Research, Ministry of Health and Sports, Myanmar, (Approval Number: Ethics/DMR/2016/099), the Science and Medical Delegated Ethics Review Committee, The Australian National University, Australia (Protocol: 2016/406), and QIMR Berghofer Human ethics committee (P1271). A waiver of consent for reanalysis of these samples for other parasites was granted by the QIMRB Human ethics committee in 2025 (P1271). In each of the selected schools, the study team conducted a meeting with parents or guardians of the fifth graders to explain the study and obtain written consent from parents or guardians for students participating in the study.

In August 2016 we conducted a stool survey in schoolchildren from Phyu township, Bago Region, for soil-transmitted helminths (STH) [26]. We initially did not consider schistosomiasis due to the focus of the grant on the STH. Notably, strongyloidiasis which has been called the most neglected of the neglected tropical diseases was only formally added to the STH grouping by the WHO in 2024 and was overlooked. The development of a sensitive qPCR assay for *A. ceylanicum* in 2017 has further helped identification of this important zoonotic helminth [27].

**Fig 3:**
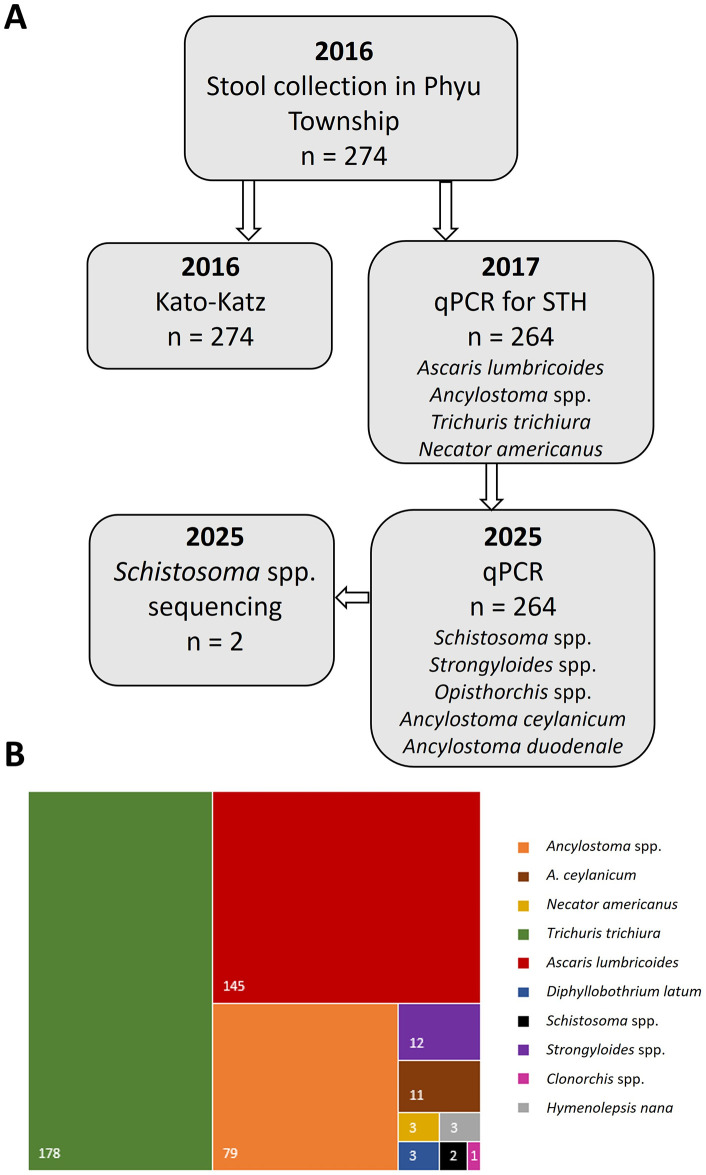
A) Flow diagram showing timing of sample collection, Kato-Katz, initial qPCR assay and more recent follow up assay and attempted sequencing in 2025 B) Total number of helminth detections by KK and qPCR assays.

Stool samples were collected from 274 schoolchildren and examined by KK for any helminth infection; 264 samples were also tested by real-time PCR (qPCR) for STH (*Ascaris lumbricoides, Ancylostoma spp., Necator americanus, Trichuris trichiura*). Prevalence of STH was high with 78.8% of schoolchildren infected by at least one STH by qPCR, and 33.3% by KK [26] (**Fig 3**). A single sample was identified by KK to be positive for *Schistosoma* spp. and another for *Strongyloides* spp., but was not reported at the time. Other helminths also identified by KK but not previously reported are *Diphyllobothrium latum* (n = 3), *Clonorchis sinensis* (n = 1), and *Hymenolepsis nana* (n = 3) (**Fig 3**).

Here, we detail the results of new qPCR assays for further helminth detection. Stool samples had previously undergone DNA extraction and qPCR for STH in 2017 [26] and it was these DNA extracts that we then revisited to test additionally for schistosomiasis using two different assays capable of detecting both *S. japonicum* and *S. mekongi* [8,28], and additionally ran qPCR assays to detect *Strongyloides* spp. [29] and *S. stercoralis* [30], *Opisthorchis viverrini* [31], and a duplex qPCR for *A. duodenale* and *A. ceylanicum* [27] (**Fig 3**)*,* PCRs were performed as per the original paper methods, with some changes. GoTaq Probe qPCR mastermix (Promega) was used for qPCR, and all probes were ordered as FAM (Integrated DNA Technologies). We also re-ran the two *Schistosoma* spp. samples for *T. trichiura* and *A. lumbricoides* as per the previous paper to test sample degradation, noting that both samples had previously been positive for these parasites. Internal controls were not used in the recent assays.

## Results

Of the 264 samples tested by qPCR, two were positive for *Schistosoma* using one of the assays, including the KK positive sample; twelve samples were positive for *Strongyloides,* which were not the same as the positive sample detected by KK, and eleven samples were positive for *A. ceylanicum*. No samples were positive for *O. viverrini* or *A. duodenale*. Full results can be found in S1 File. We attempted Big Dye sequencing of the *Schistosoma* amplicons, however our attempts failed (no visible bands on gel), highlighting limitations in template quality or primer-target mismatch. The average cycle threshold (ct) scores for both positive samples were 36.02 and 30.56. We also attempted to use generic trematode primers that we know amplify *Schistosoma* spp. DNA but were also unsuccessful [29], making low template quality the most likely cause of failure to sequence. These two *Schistosoma* spp. positive samples were previously positive for *T. trichiura* and *A. lumbricoides* and were re-tested to test the hypothesis that low template quality was causing issues with sequencing [26]. Neither sample was positive upon re-test in 2026 for *T. trichiura*, and only one was positive for *A. lumbricoides* with a ct of 32.42, 10 cycles higher than it had originally tested at, further confirming degradation of the DNA.

## Discussion

These molecular findings, though limited in number, reinforce evidence that schistosomiasis may be more widespread in Myanmar than previously acknowledged. Phyu and Shwegyin townships are roughly 100 km from each other, making local transmission in Bago Region likely. Inle Lake is farther away, about 400 km from Phyu, while the Rakhine mountain range and poor road conditions present a notable barrier between Phyu and the capital city of Rakhine State, which is approximately 800 km away. The inability to sequence the amplicon prevents species identification; clarifying whether infections are due to *S. japonicum*, *S. mekongi*, or other species is essential for guiding control strategies. We also cannot rule out multiple strains occurring, or that a specific species or strain is occurring in Myanmar as occurs in Malaysia (*S. malayensis*). The age of the DNA samples, although stored in -20°C since the original study, means that the DNA may have degraded over time leading to lower detection. Samples had undergone a minimum of three freeze-thaw cycles, and there were several occasions of the freezer malfunctioning over the intervening years which may have led to additional full or partial thaws. Additionally, just over 31% of samples had less than 200mg of stool for DNA extraction which also decreases the sensitivity of qPCR (S1 File). The two primer assays used for schistosomiasis were originally designed for *S. japonicum* detection but also show evidence of amplifying *S. mekongi* when testing specificity [8,28]. The first assay [8] which detected zero positive cases is designed on the ribosomal 28 large subunit (28*LSU*) (**Fig 1**), while the second assay [28] that detected the two positives is designed on the a repetitive tandem sequence of *S. japonicum.* The latter was previously shown a higher sensitivity than other published *S. japonicum* assays, indicating a higher copy number of the tandem repeat than targets of these other assays including mitochondrial gene NADH1, ribosomal 16S rRNA, and a putative DNA phot-lyase gene [28,32]. These sequences are also shorter, which can improve detection in cases where DNA may be more fragmented due to low template quality. The SjTR1 assay has an amplicon length of 80 bp, including primers, and a reported limit of detection (LOD) of 200 attograms for *S. japonicum* DNA [28]. The Sme assay, which did not identify any *S. mekongi* in this study, has an amplicon length of 264 bp which is quite long for a qPCR assay and could also result in the lower assay sensitivity observed here. The Sme assay does not have a stated LOD. To address this we ran a ten-fold serial dilution of *S. mekongi* DNA to 1x10^-10^ for both assays (S2 File). The SjTR1 assay was able to detect *S. mekongi* DNA to 1x10^-8^, corresponding to 1.73 femtograms (fg) of DNA, compared to 1x10^-6^, corresponding to 173 fg of DNA for the Sme assay. The SjTR1 also had higher Cts across all dilutions compared to the Sme assay, reinforcing that the SjTR1 assay has higher sensitivity.

Two assays were run for *Strongyloides* spp. detection. The first assay targeting SSU rRNA [33] only detected one positive sample, while the second assay which identifies a repeat sequence [30], detected 12 samples as positive for *S. stercoralis*. The repeat assay is highly sensitive and highly specific for *S. stercoralis* [34], while the SSU rRNA assay can detect other *Strongyloides* spp. including the zoonotic *S. fuelleborni* [35,36]. Much like the Sme assay above, the amplicon for the SSY rRNA assay is quite long at 471 bp than that of the repeat assay which has a bp of 138. Neither assay was positive for the single sample identified as positive by KK. It should also be noted that KK is not an ideal diagnostic for strongyloidiasis and this may be a mis-diagnosis [37]. This result indicates additional hidden burdens that warrant integrated surveillance, and is line with a previous survey which identified a *Strongyloides* spp. prevalence of 5.7% (n = 703) in three villages in Yangon and Ayeyarwady regions by agar plate culture [38]. Displaced populations and deteriorating water and sanitation infrastructure may be facilitating the transmission and persistence of parasitic infections, including schistosomiasis and strongyloidiasis.

The duplex for *Ancylostoma* speciation did not identify as many positives as the first *Ancylostoma* spp. qPCR assay performed in the initial study [26], and none were positive for *A. duodenale* specifically. This may again be due to decline in DNA template quality over time. The identification of the zoonotic *A. ceylanicum* in Myanmar is in line with detection in other countries within Asia [39–43], cementing that this species is a major parasite of humans in Asia. Dogs are considered the major reservoir host of this species worldwide although it has also been identified in cats and wild canid species, and is the only animal hookworm species where patent infections have been identified [43,44]. *A. ceylanicum* infections have previously been identified in Myanmar with positive samples from Bago region and Mon state, which is just South of Bago region [45]. With the high prevalence of dogs, cats, and other canids in Asia *A. ceylanicum* transmission risk is high and it is crucial that animal health and treatment as well as environmental contamination with parasite eggs is taken into consideration in traditional STH control programs as part of a One Health approach. As previously mentioned Asian schistosome species are also zoonotic, with a range of definitive mammalian hosts and reliance on a molluscan intermediate host making One Health control for schistosomes an absolute must.

Snail survey and environmental DNA (eDNA) detection to identify the intermediate host would be invaluable in determining which species is causing infection as well as providing parasite samples (from infected snails) for sequencing. The intermediate snail hosts for *S. japonicum* (*O. hupensis*) and *S. malayensis* (*Robertsiella* spp.), and *S. mekongi* (*N. aperta*) have very different habitats with the *O. hupensis* and *Robertsiella* spp. snails present in rice paddies, streams, and other slow-moving water sources with vegetation along the sides while *N. aperta* live specifically in the Mekong river, a fast-moving river, on the underside of rocks. The snail intermediate host in Myanmar for *S. japonicum* or *S. mekongi* is currently unknown. Identifying the *Schistosoma* spp. causing human infection would help narrow down potential snail hosts. Knowing the snail intermediate host will be crucial for control efforts.

Alongside snail surveys and eDNA detection, human and animal host sampling should also be performed to help fully elucidate the epidemiology of schistosomiasis in Myanmar. eDNA could be used for initial mapping of sites, allowing then for more targeted approaches to areas where parasite DNA has been identified. Although the case numbers are small, the findings are epidemiologically significant—suggesting cryptic transmission with potential for focused regional spread. We also recommend to those performing similar studies in endemic regions to look beyond the focus of the project, whether that be on STH or schistosomiasis or other specific species, and where time and funds allow, to look at identification of all possible parasites in a sample. Doing this early allows for limited DNA degradation and gives the complete picture of helminth ‌‌epidemiology in endemic regions which so often exhibit high polyparasitism.

## Supporting information

S1 File This file presents the full qPCR results for Myanmar samples, including cycle threshold (Ct) values and corresponding positive/negative (P/N) calls for all helminth assays. Column A lists the sample number, and Column B provides notes on sample preparation. Columns C and D contain the Ct values and P/N results, respectively, for *Ascaris lumbricoides* qPCR, while Columns E and F report the same for *Trichuris trichiura*. Columns G and H present Ct values and P/N outcomes for *Ancylostoma* spp., and Columns I and J for *Necator americanus*. Columns K and L contain Ct values and P/N results for *Strongyloides* spp. using the Llewellyn assay, and Columns M and N provide the corresponding data for *Strongyloides stercoralis* using the Pilotte assay. Columns O and P report Ct values and P/N calls for *Schistosoma* spp. using the Halili assay, and Columns Q and R provide Ct values and P/N results for *Schistosoma* spp. using the Adisakwattana assay.
(XLSX)

S2 File This file presents the qPCR sensitivity testing results for the Adisakwattana and Halili assays using *Schistosoma mekongi* DNA as the template. It includes cycle threshold (Ct) values generated across serial dilutions of *S. mekongi* genomic DNA.
(XLSX)

## References

[1] Gordon CA, Kurscheid J, Williams GM, Clements ACA, Li Y, Zhou X-N, et al. Asian Schistosomiasis: Current Status and Prospects for Control Leading to Elimination. Trop Med Infect Dis. 2019;4(1):40. doi: 10.3390/tropicalmed4010040 30813615 PMC6473711

[2] McManus DP, Dunne DW, Sacko M, Utzinger J, Vennervald BJ, Zhou XN. Schistosomiasis. Nat Rev Dis Primers. 2018;4(1):13. 30093684 10.1038/s41572-018-0013-8

[3] He YX, Salafsky B, Ramaswamy K. Host--parasite relationships of Schistosoma japonicum in mammalian hosts. Trends Parasitol. 2001;17(7):320–4. doi: 10.1016/s1471-4922(01)01904-3 11423374

[4] Sayasone S, Khattignavong P, Keomalaphet S, Prasayasith P, Soundala P, Sannikone S, et al. Low Prevalence of Schistosoma mekongi Infection and High Prevalence of Other Helminth Infections among Domestic Animals in Southern Lao People’s Democratic Republic. Trop Med Infect Dis. 2023;8(7):372. doi: 10.3390/tropicalmed8070372 37505668 PMC10384648

[5] Sayasone S, Mak TK, Vanmany M, Rasphone O, Vounatsou P, Utzinger J, et al. Helminth and intestinal protozoa infections, multiparasitism and risk factors in Champasack province, Lao People’s Democratic Republic. PLoS Negl Trop Dis. 2011;5(4):e1037. doi: 10.1371/journal.pntd.0001037 21532735 PMC3075221

[6] Blair D, van Herwerden L, Hirai H, Taguchi T, Habe S, Hirata M, et al. Relationships between Schistosoma malayensis and other Asian schistosomes deduced from DNA sequences. Mol Biochem Parasitol. 1997;85(2):259–63. doi: 10.1016/s0166-6851(96)02827-7 9106199

[7] Chuah C, Gobert GN, Latif B, Heo CC, Leow CY. Schistosomiasis in Malaysia: A review. Acta Trop. 2019;190:137–43. doi: 10.1016/j.actatropica.2018.11.012 30448471

[8] Adisakwattana P, Yoonuan T, Phuphisut O, Poodeepiyasawat A, Homsuwan N, Gordon CA, et al. Clinical helminthiases in Thailand border regions show elevated prevalence levels using qPCR diagnostics combined with traditional microscopic methods. Parasit Vectors. 2020;13(1):416. doi: 10.1186/s13071-020-04290-0 32787935 PMC7425172

[9] Greer GJ, Ow-Yang CK, Yong HS. Schistosoma malayensis n. sp.: a Schistosoma japonicum-complex schistosome from Peninsular Malaysia. J Parasitol. 1988;74(3):471–80. doi: 10.2307/3282058 3379527

[10] Ajjampur SSR, Sarkar R, Bradbury RS. Human passage of Schistosoma incognitum, Tamil Nadu, India, and review of autochthonous schistosomiasis, South Asia. Emerg Infect Dis. 2024;30(6):1236–9. 38782022 10.3201/eid3006.231641 PMC11138999

[11] Soe HZ, Oo CC, Myat TO, Maung NS. Detection of Schistosoma Antibodies and exploration of associated factors among local residents around Inlay Lake, Southern Shan State, Myanmar. Infect Dis Poverty. 2017;6(1):3. doi: 10.1186/s40249-016-0211-0 28245867 PMC5331719

[12] Diagnostic automation/ Cortex diagnostics. AccuDiag Schistosoma IgG. https://diagnosticautomationinc.com/AccuDiag%E2%84%A2-Schistosoma-IgG----------------_p_1652.html 2025.

[13] Reliefweb. WHO’s field visit reports over 400 schistosomiasis cases in Rakhine State. https://reliefweb.int/report/myanmar/who-s-field-visit-reports-over-400-schistosomiasis-cases-rakhine-state 2018.

[14] Kato-Hayashi N, Kirinoki M, Iwamura Y, Kanazawa T, Kitikoon V, Matsuda H, et al. Identification and differentiation of human schistosomes by polymerase chain reaction. Exp Parasitol. 2010;124(3):325–9. doi: 10.1016/j.exppara.2009.11.008 19948171

[15] Han K, Wai K, Htun MW, Aye K, Latt A, Chi Aung San N. Molecular verification of Schistosoma mekongi infection in ShweKyin Township, Myanmar. 2017.

[16] Han KT, Wai KT, Aye KH, Kyaw KW, Maung WP, Oo T. Emerging neglected helminthiasis and determinants of multiple helminth infections in flood-prone township in Myanmar. Trop Med Health. 2019;47:1. doi: 10.1186/s41182-018-0133-6 30787669 PMC6318856

[17] Bawm S, Khaing NHE, Win SY, Thein SS, Khaing Y, Thaw YN, et al. Morphological and molecular identification of trematode cercariae related with humans and animal health in freshwater snails from a lake and a dam in Myanmar. Parasitol Res. 2022;121(2):653–65. doi: 10.1007/s00436-022-07428-4 35032219

[18] Kirinoki M, Hu M, Yokoi H, Kawai S, Terrado R, Ilagan E, et al. Comparative studies on susceptibilities of two different Japanese isolates of Oncomelania nosophora to three strains of Schistosoma japonicum originating from Japan, China, and the Philippines. Parasitology. 2005;130(Pt 5):531–7. doi: 10.1017/s0031182004006924 15991496

[19] Cross JH, Zaraspe G, Lu SK, Chiu KM, Hung HK. Susceptibility of Oncomelania hupensis subspecies to infection with geographic strains of Schistosoma japonicum. Southeast Asian J Trop Med Public Health. 1984;15(2):155–60. 6505785

[20] Bunnag T, Impand P, Sornmani S. Schistosoma japonicum-like infection in Phichit province, northern Thailand: a case report. Southeast Asian J Trop Med Public Health. 1986;17(2):189–93. 3097834

[21] Bunnag T, Thirachandra S, Impand P, Vorasanta P, Imlarp S. Schistosoma incognitum and its zoonotic potential role in Phitsanulok and Phichit provinces, northern Thailand. Southeast Asian J Trop Med Public Health. 1983;14(2):163–70. 6635754

[22] Carney WP, Brown RJ, Van Peenen PF, Purnomo, Ibrahim B, Koesharjono CR. Schistosoma incognitum from Cikurai, West Java, Indonesia. Int J Parasitol. 1977;7(5):361–6. doi: 10.1016/0020-7519(77)90060-1 924721

[23] Sripa B, Suwannatrai AT, Sayasone S, Do DT, Khieu V, Yang Y. Current status of human liver fluke infections in the Greater Mekong Subregion. Acta Trop. 2021;224:106133. doi: 10.1016/j.actatropica.2021.106133 34509453

[24] Das M, Agrawal MC. Experimental infection of rhesus monkeys with Schistosoma incognitum and Orientobilharzia dattai. Vet Parasitol. 1986;22(1–2):151–5. doi: 10.1016/0304-4017(86)90018-x 3788021

[25] Dutt SC. Susceptibility of Macaca mulatta to Schistosoma incognitum, with observations on pathology of the infection. Indian J Med Res. 1967;55(11):1173–80. 4966486

[26] Aung E, Han KT, Gordon CA, Hlaing NN, Aye MM, Htun MW, et al. High prevalence of soil-transmitted helminth infections in Myanmar schoolchildren. Infect Dis Poverty. 2022;11(1):28. doi: 10.1186/s40249-022-00952-6 35272701 PMC8908594

[27] Papaiakovou M, Pilotte N, Grant JR, Traub RJ, Llewellyn S, McCarthy JS, et al. A novel, species-specific, real-time PCR assay for the detection of the emerging zoonotic parasite Ancylostoma ceylanicum in human stool. PLoS Negl Trop Dis. 2017;11(7):e0005734. doi: 10.1371/journal.pntd.0005734 28692668 PMC5519186

[28] Halili S, Grant JR, Pilotte N, Gordon CA, Williams SA. Development of a novel real-time polymerase chain reaction assay for the sensitive detection of Schistosoma japonicum in human stool. PLoS Negl Trop Dis. 2021;15(10):e0009877. doi: 10.1371/journal.pntd.0009877 34695134 PMC8568117

[29] Bowles J, Hope M, Tiu WU, Liu X, McManus DP. Nuclear and mitochondrial genetic markers highly conserved between Chinese and Philippine Schistosoma japonicum. Acta Trop. 1993;55(4):217–29. doi: 10.1016/0001-706x(93)90079-q 8147278

[30] Pilotte N, Papaiakovou M, Grant JR, Bierwert LA, Llewellyn S, McCarthy JS, et al. Improved PCR-Based Detection of Soil Transmitted Helminth Infections Using a Next-Generation Sequencing Approach to Assay Design. PLoS Negl Trop Dis. 2016;10(3):e0004578. doi: 10.1371/journal.pntd.0004578 27027771 PMC4814118

[31] Suksumek N, Leelawat K, Leelawat S, Russell B, Lek-Uthai U. TaqMan real-time PCR assay for specific detection of Opisthorchis viverrini DNA in Thai patients with hepatocellular carcinoma and cholangiocarcinoma. Exp Parasitol. 2008;119(2):217–24. doi: 10.1016/j.exppara.2008.01.018 18329641

[32] Papaiakovou M, Cimino RO, Pilotte N, Dunn J, Littlewood DTJ, Williams SA, et al. Comparison of multi-parallel quantitative real-time PCRs targeting different DNA regions and detecting soil-transmitted helminths in stool. Parasit Vectors. 2024;17(1):390. doi: 10.1186/s13071-024-06464-6 39272159 PMC11397029

[33] Llewellyn S, Inpankaew T, Nery SV, Gray DJ, Verweij JJ, Clements ACA, et al. Application of a Multiplex Quantitative PCR to Assess Prevalence and Intensity Of Intestinal Parasite Infections in a Controlled Clinical Trial. PLoS Negl Trop Dis. 2016;10(1):e0004380. doi: 10.1371/journal.pntd.0004380 26820626 PMC4731196

[34] Haidamak J, Zhao H, Watts MR, Horwood PF, Greenhill AR, Mutombo PN. Five ways to find a parasite: Comparison of five molecular assays for the detection of Strongyloides spp J Clin Microbiol. 2026.

[35] Zhao H, Mutombo PN, Mintsa-Nguema R, Nkoghe D, Atsame J, Watts M, et al. Zoonotic Soil-Transmitted Helminth Infections among Humans, Gabon. Emerg Infect Dis. 2025;31(10):2029–33. doi: 10.3201/eid3110.250816 41017110 PMC12483102

[36] Zhao H, Haidamak J, Noskova E, Ilik V, Pafčo B, Ford R. Insights into infant strongyloidiasis, Papua New Guinea. Emerg Infect Dis. 2025;31(9):1793–801. 40867023 10.3201/eid3109.241923 PMC12407202

[37] Gordon CA, Utzinger J, Muhi S, Becker SL, Keiser J, Khieu V. Strongyloidiasis. Nature Reviews Disease Primers. 2024;10(6). 10.1038/s41572-023-00490-x 38272922

[38] Aung MPPTHH, Hino A, Oo KM, Win KK, Maruyama H, Htike WW, et al. Prevalence and associated risk factors of Strongyloides stercoralis infection in Lower Myanmar. Trop Med Health. 2018;46:43. doi: 10.1186/s41182-018-0126-5 30598622 PMC6299610

[39] Kaya D, Yoshikawa M, Nakatani T, Tomo-Oka F, Fujimoto Y, Ishida K, et al. Ancylostoma ceylanicum hookworm infection in Japanese traveler who presented chronic diarrhea after return from Lao People’s Democratic Republic. Parasitol Int. 2016;65(6 Pt A):737–40. doi: 10.1016/j.parint.2016.07.001 27450724

[40] George S, Kaliappan SP, Kattula D, Roy S, Geldhof P, Kang G, et al. Identification of Ancylostoma ceylanicum in children from a tribal community in Tamil Nadu, India using a semi-nested PCR-RFLP tool. Trans R Soc Trop Med Hyg. 2015;109(4):283–5. doi: 10.1093/trstmh/trv001 25618132

[41] Inpankaew T, Schär F, Dalsgaard A, Khieu V, Chimnoi W, Chhoun C, et al. High prevalence of Ancylostoma ceylanicum hookworm infections in humans, Cambodia, 2012. Emerg Infect Dis. 2014;20(6):976–82. doi: 10.3201/eid2006.131770 24865815 PMC4036766

[42] Phosuk I, Intapan PM, Thanchomnang T, Sanpool O, Janwan P, Laummaunwai P, et al. Molecular detection of Ancylostoma duodenale, Ancylostoma ceylanicum, and Necator americanus in humans in northeastern and southern Thailand. Korean J Parasitol. 2013;51(6):747–9. doi: 10.3347/kjp.2013.51.6.747 24516284 PMC3916468

[43] Traub RJ. Ancylostoma ceylanicum, a re-emerging but neglected parasitic zoonosis. Int J Parasitol. 2013;43(12–13):1009–15. doi: 10.1016/j.ijpara.2013.07.006 23968813

[44] Liu Y, Zheng G, Alsarakibi M. The zoonotic risk of Ancylostoma ceylanicum isolated from stray dogs and cats in Guangzhou, South China. BioMed Research International. 2014;2014:208759. 24877068 10.1155/2014/208759 PMC4024403

[45] Pa Pa Aung W, Htoon TT, Tin HH, Sanpool O, Jongthawin J, Sadaow L, et al. First Molecular Identifications of Necator americanus and Ancylostoma ceylanicum Infecting Rural Communities in Lower Myanmar. Am J Trop Med Hyg. 2017;96(1):214–6. doi: 10.4269/ajtmh.16-0610 28077747 PMC5239696

